# The Half Life of Stress: Caregiver Stress Increases Next-Day Severity of Behavioral Symptoms of Dementia

**DOI:** 10.1093/geroni/igaa057.815

**Published:** 2020-12-16

**Authors:** Carolyn Pickering, Frank Puga, Danny Wang, Maria Yefimova

**Affiliations:** 1 University of Alabama at Birmingham, Birmingham, Alabama, United States; 2 The University of Alabama at Birmingham, Birmingham, Alabama, United States; 3 VA Palo Alto Healthcare System, Redwood City, California, United States

## Abstract

The progressively lowered stress threshold theory posits that behavioral symptoms of dementia (BSD) are reactions to environmental and care related stressors. In line with this theory, this study tests whether the stress experienced the caregiver impacts BSD expression. Caregivers to persons with dementia recruited online completed daily diary surveys for 21 days reporting on daily contextual and environmental factors of caregiving life. Using multi-level modeling of diaries (n=911) nested within participants (N=51), two daily caregiver stressors (conflict with someone other than the person with dementia, stress about own personal health) were examined as predictors of BSD. The outcome of BSD was measured as severity (a rating on a Likert scale of 1-5 that was then person-centered to represent deviations from average severity). Covariates in the model included relationship type, caregiver age, as well as stressors to the care recipient known to increase BSD. On days when a caregiver had a conflict with others the severity of the care recipient’s BSD increased by 1.44 points from average (p<.001, CI 1.05-1.75). In a time-lagged model, we observe that a caregiver having a conflict yesterday impacts today’s BSD severity by an increase of 0.43 points from average (p = 0.027, CI 0.05-0.81). Caregivers’ stress about their own health did not have a significant impact on the same day or next day BSD severity rating. These findings demonstrate that the well-being of the caregiver has measurable immediate impacts on the well-being of the person living with dementia, and suggests not all stress is equal.

